# Supplementary material for: Automated multilabel diagnosis on electrocardiographic images and signals
Source: Nat Commun. 2022 Mar 24;13:1583. doi: 10.1038/s41467-022-29153-3 (PMC8948243; doi:10.1038/s41467-022-29153-3)

ONLINE SUPPLEMENT

Sangha V, Khera R, et al. Automated Multilabel Diagnosis on Electrocardiographic Images and Signals

TABLE OF CONTENTS

SUPPLEMENTARY METHODS 2

Signal Model Parameters 2

SUPPLEMENTARY TABLES 3

Supplementary Table 1: Dataset Characteristics 3

Supplementary Table 2: Class Balancing Weighting Scheme for Image and Signal-Based Models. 4

Supplementary Table 3: Class Weighted Mean Performances of Real-World Model on Subsets of Held-Out Test Set with Noise Effects Introduced 5

Supplementary Table 4: Performance of image model on standard and alternate format images in the held-out test set. 6

Supplementary Table 5: Performance of image model on standard and alternate format images in the cardiologist-validated test set 7

Supplementary Table 6: Performance of signal models with and without peak morphology inputs on the held-out test set 8

Supplementary Table 7: Performance of signal models with and without peak morphology inputs on the cardiologist-validated test set 9

SUPPLEMENTARY FIGURES 10

Supplementary Figure 1: Efficientnet-B3 Architecture used for the Image Model, ... 10

Supplementary Figure 2: Custom Inception Architecture used for the Signal Model..... 11

Supplementary Figure 3: Confusion Matrices for Signal Model Predictions. 12

Supplementary Figure 4: Representative Gradient Weighted-Class Activation Maps. .. 13

SUPPLEMENTARY METHODS

Signal Model Parameters

Each convolution was padded so that input and output size was the same and was followed by a batch normalization layer and a Rectified Linear Unit (ReLU) activation layer. Inception blocks were composed of three parallel 1D convolutional blocks with kernels of size 40, 20, and 10, which were concatenated and followed by a pooling layer. The number of channels for the convolutional layers was block dependent and increased further into the model. Batch normalization was used to rescale signals after each convolutional layer, and dropout layers were added to prevent overfitting. The model was trained with an Adagrad optimizer and learning rate of 5×10^{-3} for 10 epochs with a minibatch size of 64. Model weights were randomly initialized with Xavier initialization.

SUPPLEMENTARY TABLES

Supplementary Table 1: Dataset Characteristics

	Cardiologist Validated Test Set	Held-Out Test Set	PTBXL	Training + Validation
n	827	111412	21785	2116824
Female, n (%)	506 (61.2)	67192 (60.3)	10442 (47.9)	1276674 (60.3)
Age, mean (SD)	54.9 (16.5)	53.5 (17.5)	59.8 (17.0)	53.6 (17.4)
1dAVb, n (%)	28 (3.4)	1722 (1.5)	793 (3.6)	32724 (1.5)
RBBB, n (%)	34 (4.1)	3031 (2.7)	541 (2.5)	58520 (2.8)
LBBB, n (%)	30 (3.6)	1734 (1.6)	522 (2.4)	32943 (1.6)
SB, n (%)	16 (1.9)	1808 (1.6)	637 (2.9)	33633 (1.6)
AF, n (%)	13 (1.6)	1983 (1.8)	1507 (6.9)	37679 (1.8)
ST, n (%)	37 (4.5)	2425 (2.2)	825 (3.8)	45871 (2.2)

Supplementary Table 2: Class Balancing Weighting Scheme for Image and Signal-Based Models.

Label	Positive Class n (%)	Positive Class Weight	Negative Class Weight
Female	1276674 (60.3)	0.99456179	1.00543821
1dAVb	32724 (1.5)	1.72679131	0.27320869
RBBB	58520 (2.8)	1.58113966	0.41886034
LBBB	32943 (1.6)	1.72534378	0.27465622
SB	33633 (1.6)	1.72058533	0.27941467
AF	37679 (1.8)	1.69507613	0.30492387
ST	45871 (2.2)	1.64671409	0.35328591

Abbreviations: 1dAVB - 1st degree AV block, AF – atrial fibrillation, LBBB – left bundle branch block, RBBB – right bundle branch block, SB – sinus bradycardia, ST- sinus tachycardia.

Supplementary Table 3: Class Weighted Mean Performances of Real-World Model on Subsets of Held-Out Test Set with Noise Effects Introduced

Dataset	Accuracy	PPV	NPV	Specificity	Sensitivity	AUROC	F1	AUPRC
Standardized Subset	0.989	0.688	0.997	0.992	0.841	0.992	0.755	0.736
Black and White	0.989	0.687	0.997	0.992	0.847	0.992	0.756	0.732
Rotated	0.989	0.687	0.997	0.992	0.847	0.992	0.757	0.734
Shifted Starting Point	0.989	0.704	0.996	0.993	0.816	0.992	0.753	0.743

AUROC – Area Under Receiver Operator Characteristic Curve, AUPRC – Area Under Precision Recall Curve, PPV – Positive Predictive Value, NPV – Negative Predictive Value.

Supplementary Table 4: Performance of image model on standard and alternate format images in the held-out test set.

Test Image Format	Label	Accuracy	PPV	NPV	Specificity	Sensitivity	AUROC	F1	AUPRC
**Standard**	Male	0.865	0.817	0.898	0.875	0.849	0.934	0.833	0.905
	1dAVb	0.985	0.499	0.994	0.99	0.605	0.982	0.547	0.509
	RBBB	0.989	0.75	0.997	0.992	0.878	0.994	0.809	0.801
	LBBB	0.994	0.765	0.998	0.996	0.854	0.997	0.807	0.826
	SB	0.986	0.537	0.997	0.988	0.818	0.99	0.648	0.594
	AF	0.993	0.778	0.997	0.996	0.826	0.994	0.801	0.822
	ST	0.987	0.645	0.997	0.99	0.859	0.994	0.737	0.728
	Weighted Mean	0.989	0.672	0.997	0.992	0.817	0.992	0.735	0.725
**Alternate**	Male	0.862	0.819	0.891	0.878	0.836	0.931	0.827	0.901
	1dAVb	0.984	0.496	0.994	0.99	0.609	0.982	0.547	0.51
	RBBB	0.988	0.735	0.997	0.991	0.882	0.994	0.802	0.797
	LBBB	0.993	0.757	0.998	0.996	0.851	0.997	0.801	0.821
	SB	0.986	0.536	0.997	0.989	0.8	0.991	0.642	0.588
	AF	0.992	0.769	0.997	0.996	0.82	0.994	0.794	0.811
	ST	0.987	0.663	0.997	0.99	0.845	0.994	0.743	0.73
	Weighted Mean	0.988	0.669	0.997	0.992	0.812	0.992	0.732	0.721

Abbreviations: 1dAVB - 1st degree AV block, AF – atrial fibrillation, AUPRC – Area Under Precision Recall Curve, AUROC – Area Under Receiver Operator Characteristic Curve, LBBB – left bundle branch block, NPV – Negative Predictive Value. PPV – Positive Predictive Value, RBBB – right bundle branch block, SB – sinus bradycardia, ST- sinus tachycardia

Supplementary Table 5: Performance of image model on standard and alternate format images in the cardiologist-validated test set

Test Image Format	Label	Accuracy	PPV	NPV	Specificity	Sensitivity	AUROC	F1	AUPRC
Standard	Male	0.815	0.744	0.865	0.826	0.798	0.89	0.77	0.845
	1dAVb	0.985	0.711	0.999	0.986	0.964	0.995	0.818	0.866
	RBBB	0.989	0.903	0.992	0.996	0.824	0.995	0.862	0.897
	LBBB	1	1	1	1	1	1	1	1
	SB	0.995	0.8	1	0.995	1	0.997	0.889	0.797
	AF	0.995	0.8	0.999	0.996	0.923	0.997	0.857	0.881
	ST	0.992	0.895	0.996	0.995	0.919	0.998	0.907	0.961
	Weighted Mean	0.992	0.867	0.997	0.995	0.93	0.997	0.893	0.915
Alternate	Male	0.839	0.801	0.862	0.877	0.779	0.897	0.79	0.842
	1dAVb	0.988	0.75	0.999	0.989	0.964	0.996	0.844	0.897
	RBBB	0.993	0.938	0.995	0.997	0.882	0.993	0.909	0.859
	LBBB	0.996	0.966	0.997	0.999	0.933	1	0.949	0.99
	SB	0.99	0.667	1	0.99	1	0.997	0.8	0.834
	AF	0.999	0.929	1	0.999	1	1	0.963	0.975
	ST	0.993	0.919	0.996	0.996	0.919	0.998	0.919	0.963
	Weighted Mean	0.993	0.877	0.997	0.995	0.937	0.997	0.901	0.922

Abbreviations: 1dAVB - 1st degree AV block, AF – atrial fibrillation, AUPRC – Area Under Precision Recall Curve, AUROC – Area Under Receiver Operator Characteristic Curve, LBBB – left bundle branch block, NPV – Negative Predictive Value. PPV – Positive Predictive Value, RBBB – right bundle branch block, SB – sinus bradycardia, ST- sinus tachycardia

Supplementary Table 6: Performance of signal models with and without peak morphology inputs on the held-out test set

Model	Label	Accuracy	PPV	NPV	Specificity	Sensitivity	AUROC	F1	AUPRC
**Signal + Peak Morphology**	Male	0.704	0.594	0.829	0.641	0.8	0.803	0.682	0.737
	1dAVb	0.968	0.214	0.991	0.976	0.411	0.934	0.282	0.197
	RBBB	0.987	0.733	0.995	0.992	0.827	0.987	0.777	0.745
	LBBB	0.992	0.697	0.997	0.994	0.821	0.994	0.754	0.754
	SB	0.986	0.561	0.996	0.99	0.767	0.99	0.648	0.589
	AF	0.984	0.535	0.994	0.99	0.643	0.979	0.584	0.567
	ST	0.986	0.639	0.997	0.989	0.854	0.992	0.731	0.708
	Weighted Mean	0.984	0.584	0.995	0.989	0.738	0.981	0.649	0.615
**Signal**	Male	0.716	0.612	0.824	0.674	0.781	0.808	0.686	0.743
	1dAVb	0.97	0.238	0.991	0.979	0.427	0.939	0.306	0.224
	RBBB	0.987	0.722	0.996	0.991	0.853	0.988	0.782	0.757
	LBBB	0.992	0.696	0.997	0.994	0.837	0.994	0.76	0.764
	SB	0.987	0.571	0.996	0.991	0.762	0.991	0.653	0.599
	AF	0.985	0.585	0.993	0.992	0.625	0.98	0.604	0.603
	ST	0.986	0.638	0.997	0.989	0.858	0.993	0.732	0.71
	Weighted Mean	0.985	0.594	0.995	0.99	0.745	0.982	0.659	0.63

Abbreviations: 1dAVB - 1st degree AV block, AF – atrial fibrillation, AUPRC – Area Under Precision Recall Curve, AUROC – Area Under Receiver Operator Characteristic Curve, LBBB – left bundle branch block, NPV – Negative Predictive Value. PPV – Positive Predictive Value, RBBB – right bundle branch block, SB – sinus bradycardia, ST- sinus tachycardia

Supplementary Table 7: Performance of signal models with and without peak morphology inputs on the cardiologist-validated test set

Model	Label	Accuracy	PPV	NPV	Specificity	Sensitivity	AUROC	F1	AUPRC
**Signal + Peak Morphology**	Male	0.712	0.594	0.847	0.646	0.816	0.795	0.688	0.703
	1dAVb	0.97	0.538	0.991	0.977	0.75	0.97	0.627	0.607
	RBBB	0.99	0.842	0.997	0.992	0.941	0.994	0.889	0.881
	LBBB	0.996	0.966	0.997	0.999	0.933	0.997	0.949	0.967
	SB	0.989	0.667	0.998	0.991	0.875	0.996	0.757	0.814
	AF	0.99	0.667	0.996	0.994	0.769	0.986	0.714	0.746
	ST	0.994	0.944	0.996	0.997	0.919	0.998	0.932	0.952
	Weighted Mean	0.988	0.803	0.996	0.992	0.88	0.991	0.836	0.848
**Signal**	Male	0.713	0.597	0.841	0.656	0.804	0.803	0.685	0.711
	1dAVb	0.971	0.577	0.984	0.986	0.536	0.96	0.556	0.501
	RBBB	0.99	0.882	0.995	0.995	0.882	0.997	0.882	0.928
	LBBB	0.995	1	0.995	1	0.867	0.998	0.929	0.97
	SB	0.989	0.667	0.998	0.991	0.875	0.995	0.757	0.686
	AF	0.994	1	0.994	1	0.615	0.993	0.762	0.802
	ST	0.993	0.878	0.999	0.994	0.973	0.998	0.923	0.923
	Weighted Mean	0.989	0.837	0.994	0.994	0.816	0.99	0.82	0.824

Abbreviations: 1dAVB - 1st degree AV block, AF – atrial fibrillation, AUPRC – Area Under Precision Recall Curve, AUROC – Area Under Receiver Operator Characteristic Curve, LBBB – left bundle branch block, NPV – Negative Predictive Value. PPV – Positive Predictive Value, RBBB – right bundle branch block, SB – sinus bradycardia, ST- sinus tachycardia

SUPPLEMENTARY FIGURES

Supplementary Figure 1: Efficientnet-B3 Architecture used for the Image Model,

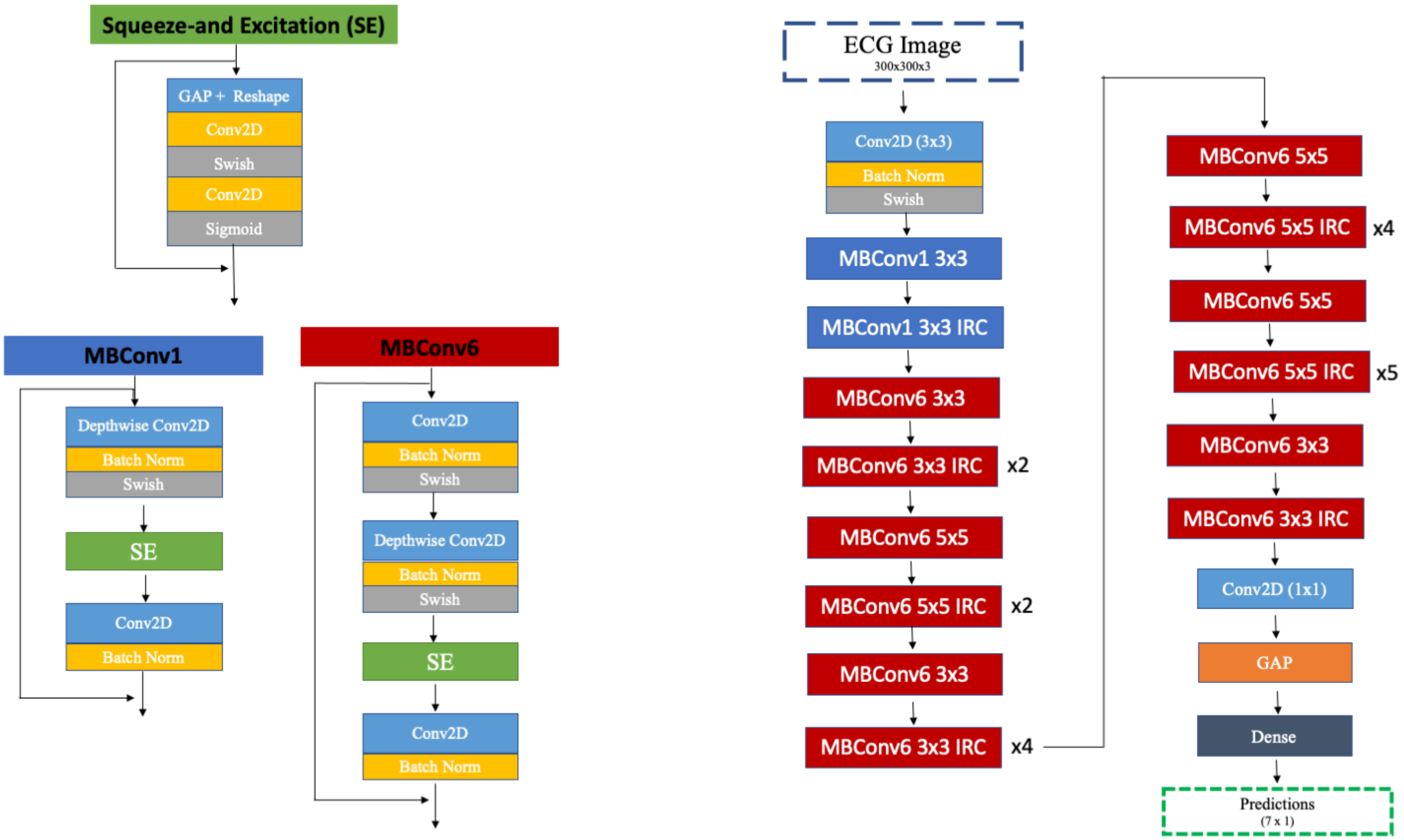

Supplementary Figure 2: Custom Inception Architecture used for the Signal Model.

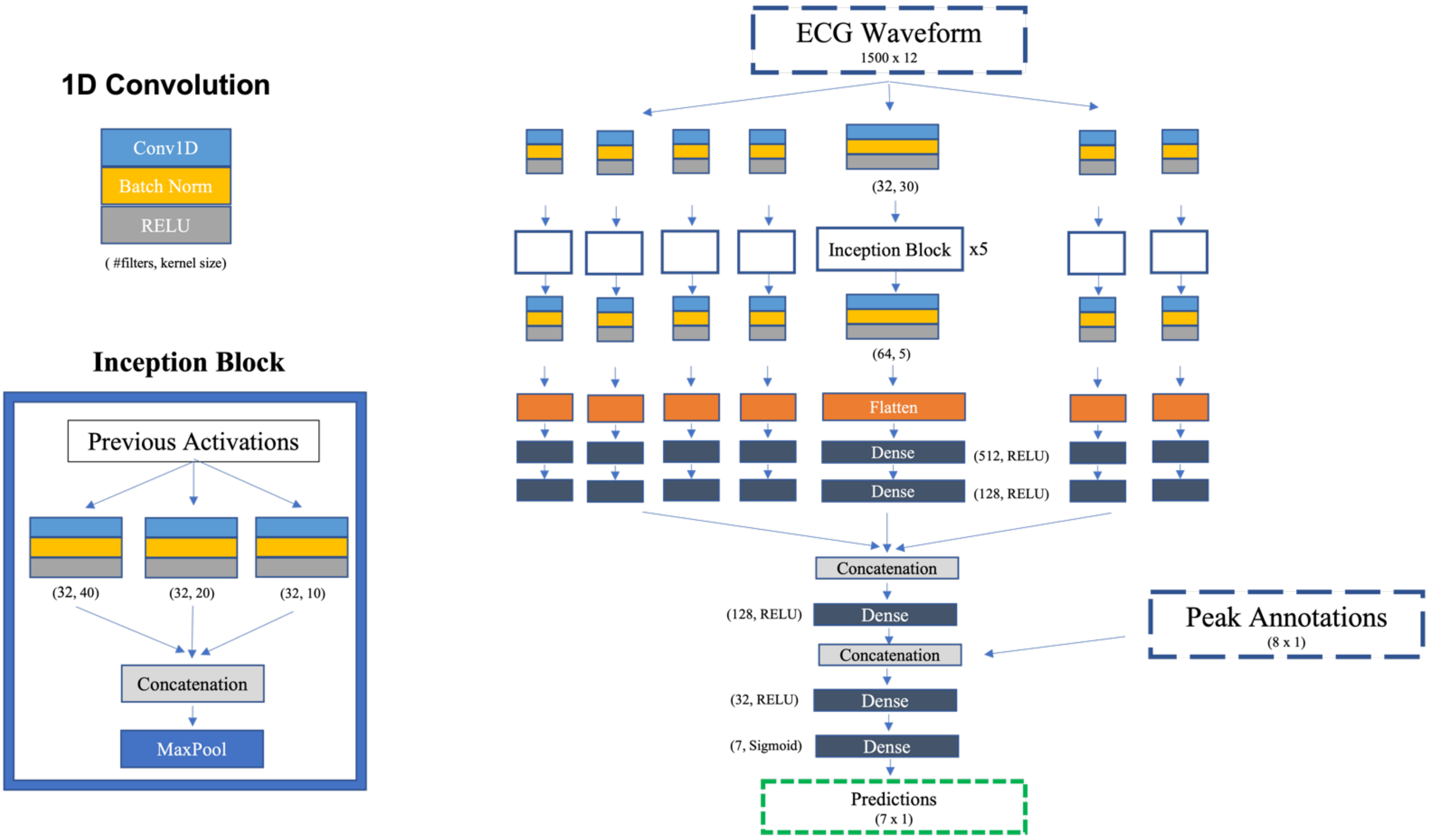

Supplementary Figure 3: Confusion Matrices for Signal Model Predictions.

Abbreviations: 1dAVB - 1st degree AV block, AF – atrial fibrillation, LBBB – left bundle branch block, RBBB – right bundle branch block, SB – sinus bradycardia, ST- sinus tachycardia.

A. Held-out Test Set

B. Cardiologist-Validated Test Set

C. PTB-XL

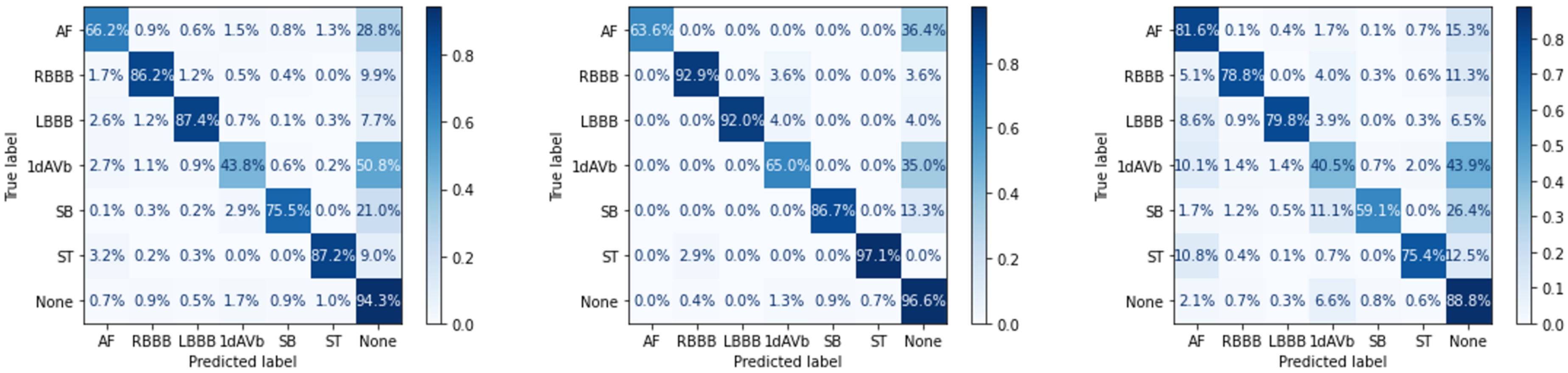

Supplementary Figure 4: Representative Gradient Weighted-Class Activation Maps.

Abbreviations: LBBB – left bundle branch block, RBBB – right bundle branch block.

A. RBBB

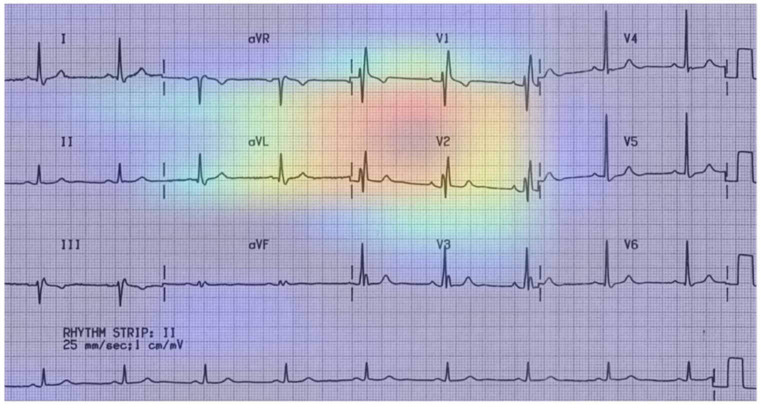

B. RBBB

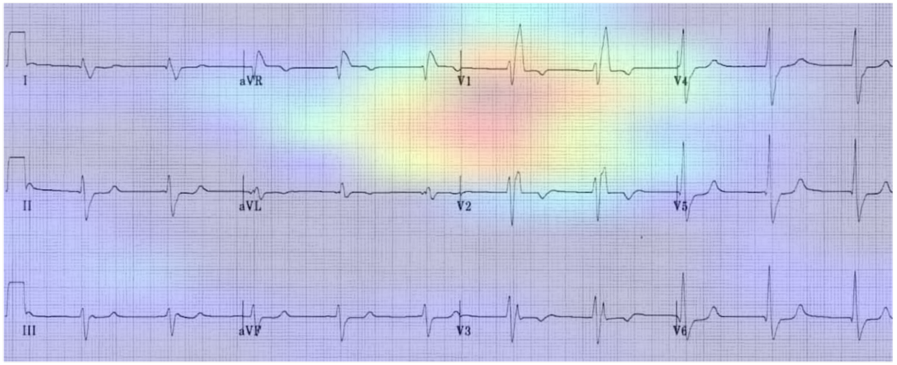

C. LBBB

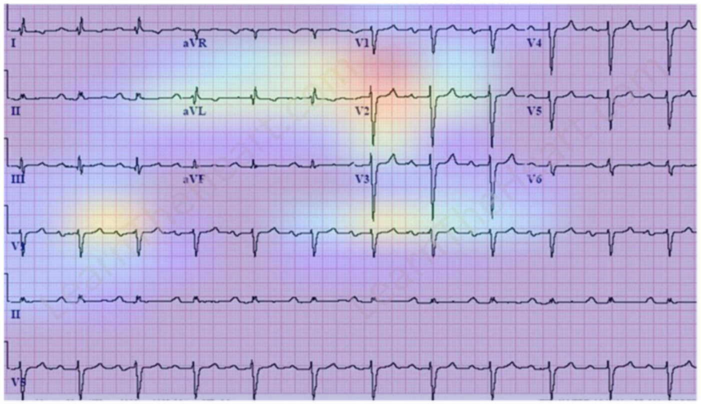

D. LBBB

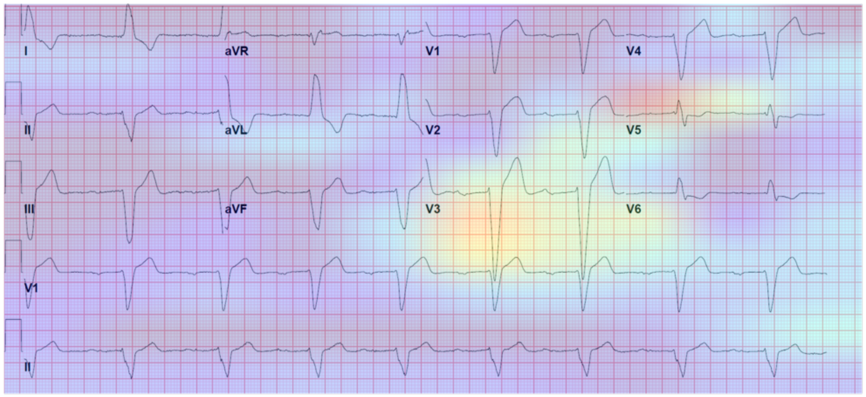

Supplement: Supplementary file 1 — Supplementary Information [file 41467_2022_29153_MOESM1_ESM.pdf]
