# Supplementary material for: Automated multilabel diagnosis on electrocardiographic images and signals
Source: Nat Commun. 2022 Mar 24;13:1583. doi: 10.1038/s41467-022-29153-3 (PMC8948243; doi:10.1038/s41467-022-29153-3)
Supplement: Supplementary file 2 — Reporting Summary [file 41467_2022_29153_MOESM2_ESM.pdf]

nature portfolio

Corresponding author(s): Rohan Khera, MD, MS

Last updated by author(s): Feb 25, 2022

Reporting Summary

Nature Portfolio wishes to improve the reproducibility of the work that we publish. This form provides structure for consistency and transparency in reporting. For further information on Nature Portfolio policies, see our Editorial Policies and the Editorial Policy Checklist.

Statistics

For all statistical analyses, confirm that the following items are present in the figure legend, table legend, main text, or Methods section.

n/a	Confirmed	
☐	☒	The exact sample size (n) for each experimental group/condition, given as a discrete number and unit of measurement
☐	☒	A statement on whether measurements were taken from distinct samples or whether the same sample was measured repeatedly
☐	☒	The statistical test(s) used AND whether they are one- or two-sided<br>*Only common tests should be described solely by name; describe more complex techniques in the Methods section.*
☐	☒	A description of all covariates tested
☒	☐	A description of any assumptions or corrections, such as tests of normality and adjustment for multiple comparisons
☐	☒	A full description of the statistical parameters including central tendency (e.g. means) or other basic estimates (e.g. regression coefficient) AND variation (e.g. standard deviation) or associated estimates of uncertainty (e.g. confidence intervals)
☒	☐	For null hypothesis testing, the test statistic (e.g. F, t, r) with confidence intervals, effect sizes, degrees of freedom and P value noted<br>*Give P values as exact values whenever suitable.*
☒	☐	For Bayesian analysis, information on the choice of priors and Markov chain Monte Carlo settings
☒	☐	For hierarchical and complex designs, identification of the appropriate level for tests and full reporting of outcomes
☐	☒	Estimates of effect sizes (e.g. Cohen's d, Pearson's r), indicating how they were calculated

Our web collection on statistics for biologists contains articles on many of the points above.

Software and code

Policy information about availability of computer code

Data collection	All analyses were performed using Python 3.9. The signal data was plotted using the python package, ecg-plot 0.2.8. Signal processing was performed using NeuroKit2 and Scipy 1.7.
Data analysis	Models were developed using TensorFlow 2.5 and performance characteristics were assessed using scikit-learn 1.0. The code used in the study are proprietary, and were reviewed by the Editors and reviewers during peer review. While the code are not available in a public repository, pertinent sections will be shared by the authors upon request. The model is publicly available as a web-based tool: https://www.cards-lab.org/ecgdx.

For manuscripts utilizing custom algorithms or software that are central to the research but not yet described in published literature, software must be made available to editors and reviewers. We strongly encourage code deposition in a community repository (e.g. GitHub). See the Nature Portfolio guidelines for submitting code & software for further information.

Data

Policy information about availability of data

All manuscripts must include a data availability statement. This statement should provide the following information, where applicable:

- Accession codes, unique identifiers, or web links for publicly available datasets
- A description of any restrictions on data availability
- For clinical datasets or third party data, please ensure that the statement adheres to our policy

The cardiologist-validated test set is publicly available on the link: https://zenodo.org/record/3765780#.YVIM8J5Kgl9%2F. The training data are based on the CODE study, published in Nature Communications (Volume 11, Article number: 1760 (2020)) and can be obtained from Ribeiro et al. Restrictions apply to the availability of the training set and requests to access will need to be submitted and review on an individual basis by the Telehealth Network of Minas Gerais for academic use only.

The data for PTB-XL are available from physionet.org. The test data from Lake Regional Health System and the web-based validation set will be made available from the authors upon request.

Field-specific reporting

Please select the one below that is the best fit for your research. If you are not sure, read the appropriate sections before making your selection.

☒ Life sciences ☐ Behavioural & social sciences ☐ Ecological, evolutionary & environmental sciences

For a reference copy of the document with all sections, see nature.com/documents/nr-reporting-summary-flat.pdf

Life sciences study design

All studies must disclose on these points even when the disclosure is negative.

Sample size	2.2 million ECGs from Brazil and 20 thousand from PTB-XL as external validation. The study included all available data and did not pursue a sample size calculation.
Data exclusions	The only data excluded were those without interpretable data, representing signals suggestive of cardiac activity.
Replication	The results were externally validated in data sources that were not involved in model development. This was done with 3 independent data sources. The performance of the model on these data sources were reporting using area under the receiver operating characteristic curve, area under the precision-recall curve, the F1 score and model sensitivity, specificity, and positive and negative predictive values.
Randomization	This was n/a for our observational study
Blinding	This was n/a for our observational study

Reporting for specific materials, systems and methods

We require information from authors about some types of materials, experimental systems and methods used in many studies. Here, indicate whether each material, system or method listed is relevant to your study. If you are not sure if a list item applies to your research, read the appropriate section before selecting a response.

Materials & experimental systems

n/a	Involved in the study	
☒	☐	Antibodies
☒	☐	Eukaryotic cell lines
☒	☐	Palaeontology and archaeology
☒	☐	Animals and other organisms
☒	☐	Human research participants
☒	☐	Clinical data
☒	☐	Dual use research of concern

Methods

n/a	Involved in the study	
☒	☐	ChIP-seq
☒	☐	Flow cytometry
☒	☐	MRI-based neuroimaging
